# Research on Interferometric Tilt Sensor for Vibration Isolation Platform

**DOI:** 10.3390/s25061777

**Published:** 2025-03-13

**Authors:** Weigang Bai, Wenwu Feng, Peigen Wang, Ziliang Zhang, Guoying Zhao

**Affiliations:** MOE Key Laboratory of TianQin Mission, TianQin Research Center for Gravitational Physics & School of Physics and Astronomy, Frontiers Science Center for TianQin, Gravitational Wave Research Center of CNSA, Sun Yat-sen University (Zhuhai Campus), Zhuhai 519082, China

**Keywords:** tilt-horizontal coupling, interferometric tilt sensor, vibration isolation

## Abstract

Low-frequency seismic vibrations extremely limit the performance of ground simulation facilities for space-borne gravitational wave detections, which need to be substantially suppressed. Active vibration systems are thus required. However, the tilt-translation coupling of inertial sensors strongly limits the performance of vibration isolation platforms in the low frequency range, which requires a precise measurement of the low-frequency tilt signal. This study compares two methods for the tilt signal measurement: the differential-mode method and the direct method. The differential-mode method estimates tilt signals by analyzing differential motion between two inertial sensors, while the direct method utilizes an interferometric tilt sensor (ITS) which consists of a suspended rotational beam system and an interferometer for the readout. Experimental results show that ITS achieves a lower noise floor. Its noise floor is dominated by the thermal-mechanical noise below 0.25 Hz and the readout noise of the interferometer above 0.25 Hz. The findings highlight the potential of ITS for improving the performance of vibration isolation platforms in the low-frequency range.

## 1. Introduction

Vibration isolation devices play a crucial role in ultra-precision manufacturing and measurement processes, including applications in photolithography machines [1,2,3] and particle colliders [4,5]. As science and technology continue to advance, ultra-precision devices require increasingly stringent specifications for environmental vibration control, driving the growing demand for vibration isolation systems targeted at lower frequency bands. For instance, research focuses on low-frequency vibration isolation technologies can be found in applications such as cold atom gravimeters [6], electrostatic accelerometers [7], and hull grillage metastructures [8]. To further illustrate this trend, we refer to the development of gravitational wave detection equipment. The frequency bands of first- and second-generation detectors primarily range from 100 Hz to 300 Hz and from 10 Hz to 10 kHz, respectively [9,10]. Third-generation ground-based gravitational wave detectors are designed with detection capabilities extending from 1 Hz to 10 kHz [11,12,13]. Furthermore, space-based gravitational wave detectors, such as the LISA project [14], the Taiji project [15,16,17], and the Tianqin project [18,19,20], enable more accurate low-frequency gravitational wave detection.

Low-frequency seismic vibrations extremely limit the performance of ground simulation facilities for space-borne gravitational wave detections, which need to be substantially suppressed. Active vibration systems are thus required. However, distinguishing between low-frequency horizontal and tilt motions is challenging [21] due to limitations in seismometer technology, leading to tilt-horizontal coupling that restricts the performance of vibration isolation platforms [22]. An effective approach to enhance the low-frequency range from 0.1 Hz to 1 Hz performance of these platforms involves accurately measuring the platform’s tilt signals and subtracting them to isolate horizontal motion. Various methodologies have been developed for tilt signal measurement. Matichard used differential-mode vertical signals from Trillium T240 seismometers [23]. The Virgo collaboration employs a balanced beam for the tilt measurement [24] The Advanced LIGO (aLIGO) project introduced the balanced rotational sensor (BRS) with a flexible hanging beam [25]. Caltech’s LIGO utilized an accelerometer with blade-like structures and LVDTs [26]. Paroscientific is exploring quartz rotation sensors (QRSs) with quartz pressure sensing [27]. In this paper, an interferometric tilt sensor is proposed, which is featured with a low noise floor. The ITS is integrated into a vibration isolation platform, where the performance of ITS is compared with a traditional method for the tilt signal measurement.

This paper introduces and compares two methods for measuring platform tilt signals and directly applies them to vibration isolation platforms. Our analysis commences with a theoretical description of these methods. Next, we describe the developed ITS system, including its mechanical structure, readout mechanism, and noise analysis. Finally, we conducted an experimental verification, placing three sets of tilt signal measurement devices on the same platform, including two ITS devices and two commercial Guralp 3ESPC sensors (manufactured by Guralp Systems, Reading, UK). By comparing the three sets of tilt signals pairwise, we verified the accuracy of the self-developed sensor measurement and discussed the prospects for future work in this field.

## 2. Tilt Signal Measurement Method

This paper introduces two methods for measuring tilt signals: the differential-mode method and the direct method.

### 2.1. Differential-Mode Method

**Figure 1:**
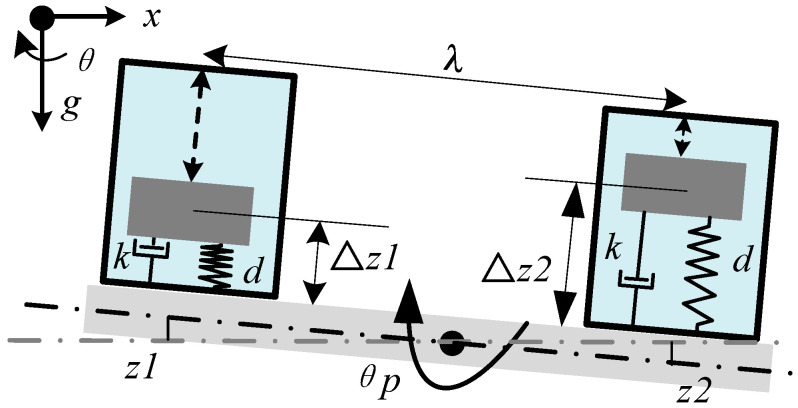
The schematic of the differential-mode method.

The differential-mode method requires two vertical inertial sensors that are placed at a specific distance in the platform, as show in Figure 1. The symbols *z*_1_ and *z*_2_ denote the vertical displacements of the platform where the inertial sensors are located; Δ*z*_1_ and Δ*z*_2_ are the relative displacements between the proof masses and the platform; *λ* represents the distance between the two proof masses; *k*_1_, *k*_2_, *d*_1_, and *d*_2_ are the stiffness and the damping coefficient of the left and right inertial sensors; *m*_1_ and *m*_2_ represent the masses of the proof mass, and *θ_p_* represents the tilt angle of the platform.

The sensitivity of the inertial sensor to the excitations can be derived as

(1) $$\frac{△z_{1}}{z_{1}}=\frac{-m_{1}s^{2}}{m_{1}s^{2}+k_{1}+d_{1}s}$$

(2) $$\frac{△z_{2}}{z_{2}}=\frac{-m_{2}s^{2}}{m_{2}s^{2}+k_{2}+d_{2}s}$$

The actual tilt signal *θ_p_* and the tilt signal *θ_ag_* estimated by the differential-method read

(3) $$\theta_{p}=\frac{z_{2}-z_{1}}{\lambda }$$

(4) $$\theta_{ag}=\frac{△z_{2}-△z_{1}}{\lambda }$$

Given two identical vertical inertial sensors, the sensitivities are the same, where *m*_1_ *= m*_2_, *k*_1_ *= k*_2_, and *d*_1_ *= d*_2_:

(5) $$\theta_{p}=\frac{△z_{2}-△z_{1}}{\lambda }\frac{ms^{2}+k+ds}{-ms^{2}}$$

(6) $$\frac{\theta_{ag}}{\theta_{p}}=\frac{-ms^{2}}{ms^{2}+k+ds}$$

**Figure 2:**
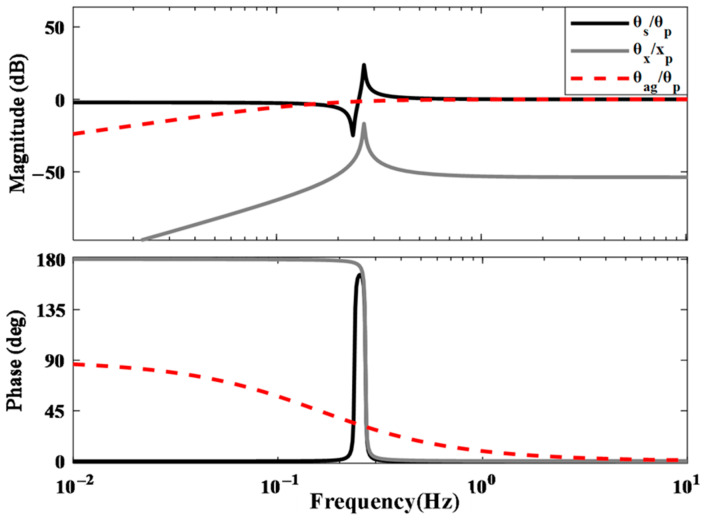
The sensitivities to tilt and translational excitations.

Substituting the parameters of the vertical inertial sensor into Equation (6) allows us to obtain the sensitivity of the differential-mode method to platform tilt, as shown in Figure 2.

In addition, the vertical inertial sensor is also sensitive to rotational excitations in practice. This happens when the deflection angle of the proof mass is not perfectly aligned with respect to the local gravity. The initial deflection angles of the proof mass in the platform’s left and right vertical inertial sensors are not identical, leading to differences in the vertical signals measured by these two vertical inertial sensors [28].

### 2.2. The Balanced Beam Method

**Figure 3:**
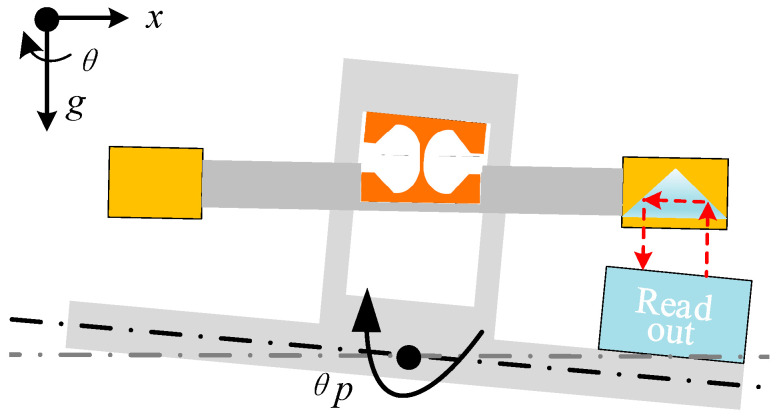
The schematic of the balanced beam method.

The tiltmeter rotates around the pivot axes. The center of mass of the balanced beam is located as close to the axis of rotation as possible, and this arrangement can decouple the influence of rotation and translation. Above the resonant frequency, the balanced beam remains horizontally inertial. The interferometer readout is fixed on the platform which measures the relative displacement between the platform and the beam. The relative displacement is divided by half the beam length to obtain the overall tilt signal of the platform, as shown in Figure 3.

We can consider the tiltmeter as a rotational spring–mass system, with the dynamic equation of motion as follows [25]:

(7) $$I\ddot{\theta }+\gamma \dot{\theta }+k(1+\frac{i}{Q})(\theta -\theta_{p})+Mg\delta \theta +M\delta {\ddot{x}}_{p}=\tau_{ex}$$

In this equation, θ is the angle between the balanced beam and the horizontal direction. $\theta_{p}$ is the angle between the experimental platform and the horizontal direction. $\tau_{ex}$ is the sum of all exterior torques. *I* is the moment of inertia of the beam. *Q* is the intrinsic quality factor of the structure. γ is the velocity damping factor. κ is the spring constant of the flexures. *M* is the mass of the balance. *g* is the gravitational acceleration. *δ* is the vertical distance from the pivot point to the center of mass of the interferometric tilt sensor (ITS). *x_p_* is the horizontal displacement of the platform. According to the relationship between angles, Equation (7) can be rearranged to obtain

(8) $$\theta_{a}=\theta -\theta_{p}=-\frac{\tau_{ex}+\omega^{2}M\delta x_{p}-(I\omega^{2}-i\gamma \omega -Mg\delta )\theta_{p}}{I\omega^{2}-i\gamma \omega -i\kappa /Q-\kappa -Mg\delta }$$

Upon analyzing Equation (8), we can divide the readout into three parts:

(9) $$\theta_{a}=\theta_{\tau }+\theta_{x}+\theta_{s}$$

(10) $$\theta_{x}=-x_{p}\frac{M\delta }{I}\frac{\omega^{2}}{\omega^{2}-i(\omega_{0}\omega /q+\omega_{0}^{2}/Q)-(\omega_{0}^{2}+\omega_{g}^{2})}$$

(11) $$\frac{\theta_{x}}{x_{p}}=-\frac{M\omega^{2}\delta }{I(\omega^{2}-i(\omega_{0}\omega /q+\omega_{0}^{2}/Q)-(\omega_{0}^{2}+\omega_{g}^{2}))}$$

(12) $$\theta_{s}=-\theta_{p}\frac{\omega^{2}-i\omega_{o}\omega /q-\omega_{g}^{2}}{\omega^{2}-i(\omega_{0}\omega /q+\omega_{0}^{2}/Q)-(\omega_{0}^{2}+\omega_{g}^{2})}$$

(13) $$\frac{\theta_{s}}{\theta_{p}}=-\frac{\omega^{2}-i\omega_{o}\omega /q-\omega_{g}^{2}}{\omega^{2}-i(\omega_{0}\omega /q+\omega_{0}^{2}/Q)-(\omega_{0}^{2}+\omega_{g}^{2})}$$

where *θ_x_* represents the angle deflection caused by platform translation, *θ_s_* represents the angle deflection caused by platform rotation, *θ_τ_* represents the angle deflection caused by external torque and is assumed to be zero, $\omega_{0}=\sqrt{k/I}$ is the resonant frequency of the structure, and $\omega_{g}=\sqrt{Mg\delta /I}$.

Substituting the tiltmeter parameters into Equations (11) and (13) allows us to obtain the sensitivities of the tiltmeter to tilt and translational excitations, as illustrated in Figure 2. The black line shows the sensitivity of the tiltmeter to platform tilt; the solid gray line shows the sensitivity of the tiltmeter to translation, and the red solid line represents the sensitivity to platform tilt under the differential-mode method. The figure clearly shows that the response of the tiltmeter to the tilt signal is significantly higher than that to the horizontal signal across the entire frequency band, facilitating better decoupling of the tilt and horizontal signals. This decoupling is essential for minimizing the tilt-horizontal coupling effect. For further theoretical and experimental details, please refer to [23]. Further comparison of the red solid line and the black solid line shows that, at low frequencies below 0.5 Hz, the sensitivity of the differential-mode method is significantly reduced compared to the balanced beam method, which is not conducive to the measurement of tilt signals.

## 3. Development of ITS

### 3.1. ITS Mechanics and Readout

**Figure 4:**
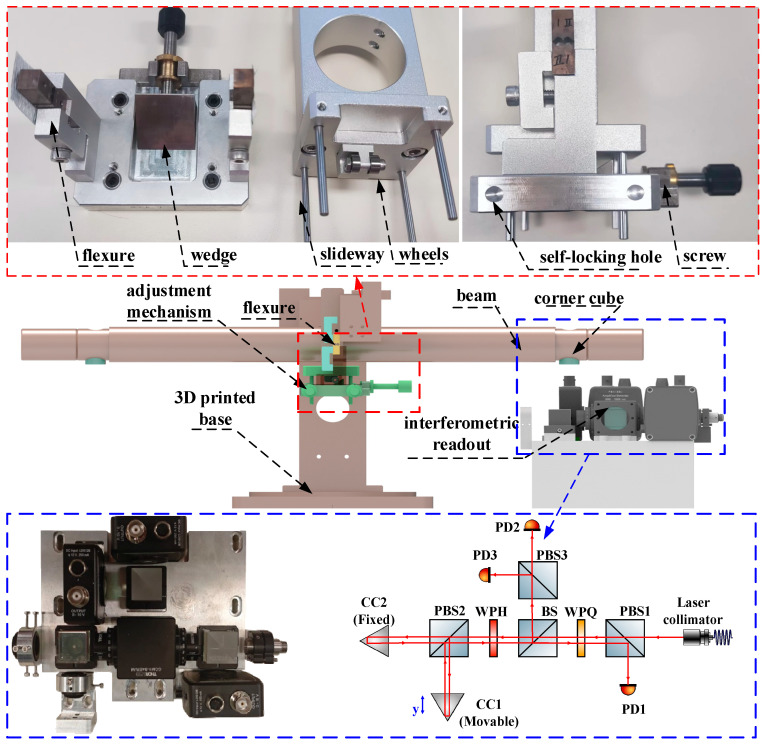
The design of the ITS.

The scheme of the proposed ITS is shown in Figure 4. The ITS is mainly composed of two subsystems: (i) a mechanical oscillator and (ii) a readout. The mechanical oscillator consists of a balance beam, a flexure, and an adjustment mechanism for tuning the center of mass of the system. The balance beam consists of an aluminum alloy tube with copper blocks fixed at both ends. The balance beam and its connectors are suspended together by a flexible structure, and the thinnest part is only 30 μm, posing challenges for processing and assembly. Therefore, the design of the notch shape of the flexure is crucial. Initially, a relatively stable semicircular notch shape was selected; however, it was prone to over-range fractures during the assembly and experimental processes. To mitigate this issue, we added a corner limiter at the end of the notch to prevent the fracture of the flexible structure, thus enabling a self-protective function. The total weight of the ITS is about 3 kg, and the balanced beam has a length of 400 mm. The torsional stiffness of the flexure is 2.5 × 10^−3^ N·m/rad. For the interferometric readout system, its working principle is similar to the one that has been used for implementing the interferometric inertial sensor.

For the readout, a homemade interferometer is chosen. The optical path of the laser interferometer includes the laser collimator, polarizing beam splitters (PBS1, PBS2, and PBS3), and the wave plate (WPH and WPQ) for optical alignment. The light is split and directed toward the movable and fixed corner cubes (CC1 and CC2), and the resulting interference patterns are detected by the photodiodes (PD1, PD2, and PD3). The signal is processed to measure the displacement with high accuracy.

The optical readout system uses corner cubes (CC1 and CC2) to reflect the lasers. The movable corner cube (CC1) is locked during the initial adjustment until the interference pattern is clear; then, it is released for precise alignment. The system ensures that the optical path remains stable during measurements, even under micro-vibrations, preventing divergence and ensuring the light path remains intact. For more details on the interferometric readout mechanism and the corresponding signal processing method, please refer to [29,30]. The system implemented in this study has a resolution of 1 $\mathrm{pm}/\sqrt{\mathrm{Hz}}$ at 1 Hz.

### 3.2. Decoupling Adjustment Mechanism Design

To mitigate the effects of tilt-horizontal coupling on the ITS, the response to horizontal displacement excitation can be derived from Equation (13) given *ω* ≫ *ω*_0_:

(14) $$\frac{\theta_{x}}{x_{p}}\approx \frac{M\delta }{I}$$

Based on the above equation, we designed a decoupling adjustment mechanism. As illustrated in Figure 4, a pair of pulleys is mounted beneath the fixed frame of the balance beam, which collaborates with a wedge block to fine-tune the distance between the balance beam’s center of mass and the suspension point. The pair of pulleys is designed to reduce friction throughout the adjustment process. The inclusion of the wedge block enhances adjustment precision by leveraging the angle between its inclined surface and the platform. The wedge block also redirects force, converting horizontal thrust into vertical lift. This design prevents direct axial loading on the flexures, protecting them from compression or tearing due to adjustment torque, while transforming rotational torque on the flexures into vertical tension.

Moreover, to precisely control the adjustment direction of the center of mass, four guide rails are installed beneath the balance beam bracket, ensuring that movements are confined to vertical displacement only. This setup allows the horizontal motion of the wedge block to be seamlessly translated into the vertical movement of the balance beam.

The decoupling adjustment mechanism fine-tunes the relative position of the center of mass with respect to the pivot point without altering the mass distribution across the balance beam. By adjusting the vertical position of the suspended component, this mechanism minimizes the impact of horizontal displacement on tilt measurements. By altering the magnitude of *δ*, this mechanism adjusts the vertical center of mass height of the suspended component and alleviates the assembly pressure on the flexures. When *δ* decreases, the procedure entails rotating a screw to elevate the wedge block, thereby lowering the height of the suspended body and its suspension point in the vertical plane, thus adjusting the vertical center of mass position. A similar procedure is followed when *δ* increases.

### 3.3. System Composition and Noise Budget

**Figure 5:**
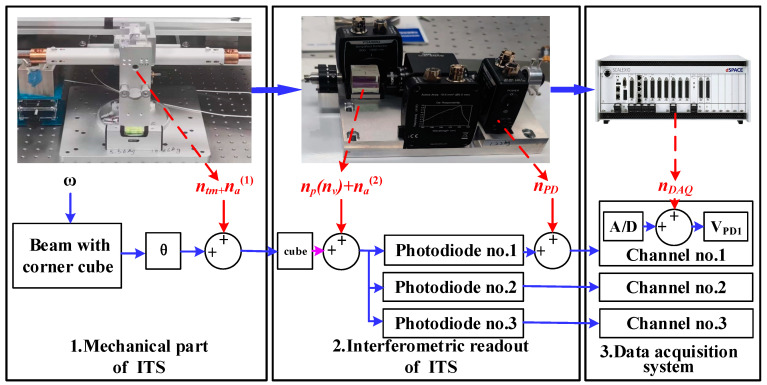
Diagram of noise contribution during measurements.

Figure 5 illustrates the noise contribution diagram for the ITS measurement process. The ITS structure is composed of three primary components: the mechanical oscillator, the interferometric readout, and the data acquisition system, which are the main sources of noise in the ITS. The diagram uses various color-coded lines to represent different aspects of the system. The main noise sources shown in the figure are as follows: *n_tm_* for thermal-mechanical noise, *n_a_* for ambient noise, and *n_v_* for laser frequency noise; the laser frequency noise was estimated based on the data from the laser user manual. Although low-frequency noise is not directly provided in the datasheets, we used available data and theoretical models to estimate the laser frequency noise in the 0.01–0.1 mHz range. *n_p_* is for phase noise concerning the laser frequency noise; *n_PD_* is for photodiode noise, and *n_DAQ_* is for data acquisition noise. Indexes (1) and (2) present the same type of noise with different values.

Mechanical Structure: the primary sources of noise are thermal-mechanical noise and environmental noise caused by airflow disturbances and temperature variations. Interferometric Readout: The noise primarily comprises phase noise due to laser frequency instability, environmental noise from optical element imperfections and misalignments in optical elements, and noise from the photodetector. It should be noted that laser intensity noise is coherently eliminated through data processing using voltage signals from three photodetectors, while environmental noise is significantly mitigated by employing thermal shields and precise alignment of optical elements; hence, these are not accounted for in the noise budget. Data Acquisition System: the predominant noise source is analog-to-digital converter (ADC) noise.

**Figure 6:**
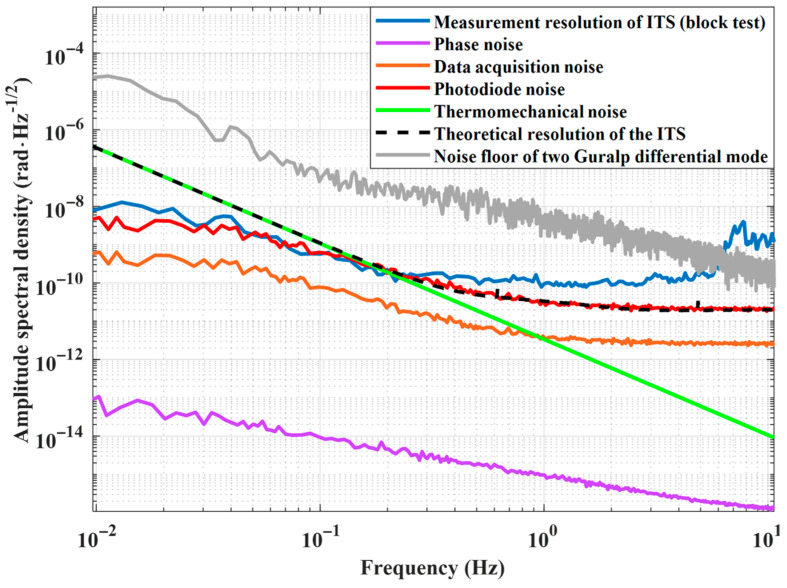
Noise budget of the ITS.

As depicted in Figure 6, the noise sources of the ITS encompass thermal-mechanical noise, phase noise, data acquisition (DAQ) noise, and photodiode (PD) noise. The equivalent angular noise curves for each noise source are presented. The theoretical ITS noise floor is primarily influenced by thermal-mechanical noise below 0.21 Hz and by PD noise above 0.21 Hz. From Figure 3, although the sensitivities to tilt excitations of both methods are identical above 0.2 Hz, ITS has a lower noise floor, allowing it to detect weaker tilt signals.

The measured ITS noise floor, however, is predominantly influenced by ground vibration noise from 0.21 Hz to 10 Hz due to the flexibility of the readout structure. When compared to the differential-mode method, it is evident that the ITS noise floor is significantly below the noise level achieved by the differential-mode method up to 5 Hz.

## 4. Experimental Verification

### 4.1. Huddle Test

**Figure 7:**
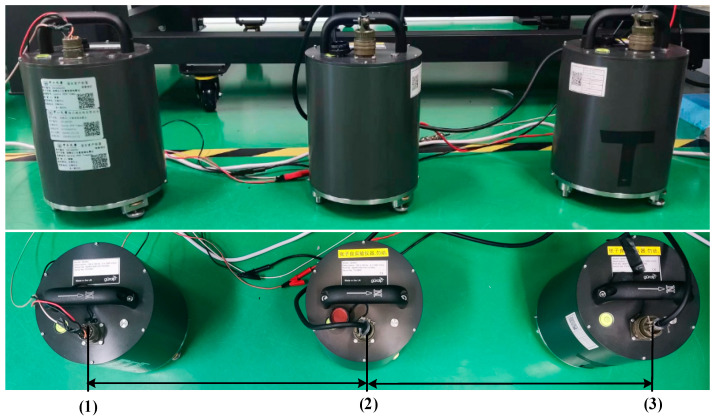
Picture of the huddle test.

We determined the noise floor of the differential-mode method through a huddle test on signals from three adjacent Guralp sensors, as depicted in Figure 7. In the huddle test, two independent sensors are used to measure the same physical signal. The signals from these sensors are subtracted, with the assumption that their noise sources are incoherent. This allows us to estimate the noise floor of the system by removing the common-mode signal and retaining only the independent noise contributions from each sensor [28,31]. The Guralp 3ESPC sensor employs a capacitive displacement readout mechanism to measure the displacement of the seismic mass. The measurement duration was set to 2000 s. The gray curve in Figure 6 illustrates the noise floor achieved using the differential-mode approach.

### 4.2. Platform

**Figure 8:**
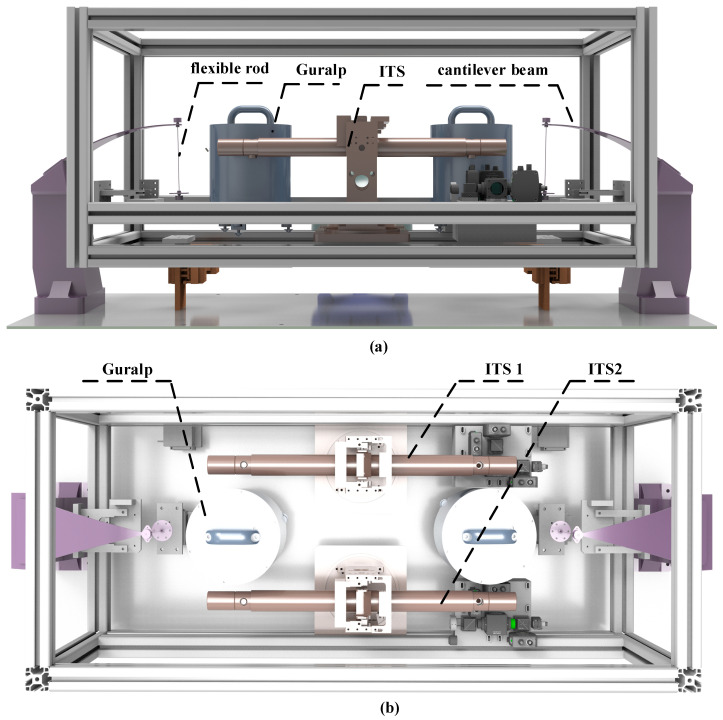
Three-dimensional diagram of the isolation platform: (**a**) platform structure and composition; (**b**) three sets of tilt signal measurement sensors.

As illustrated in Figure 8a, the passive isolation platform comprises a suspension system, two seismometers (Guralp), and two ITS devices. The experimental platform uses a passive suspension system, which is designed to support a total mass of approximately 60 kg. The key flexible components, including cantilever beams and flexible rods, provide passive isolation in both the vertical and horizontal directions. The natural frequencies for the vertical and horizontal degrees of freedom are set to 1.8 Hz and 1.32 Hz, respectively. As shown in Figure 8b, on the platform, the Guralp sensor group is located on the left and right sides of the platform’s central axis, and it is responsible for measuring the vertical motion signals at both ends of the platform; tilt signals are obtained through the differential-mode method. ITS1 and ITS2 are installed on the front and rear sides of the Guralp sensor group, with a relatively short distances among the three sensors. They independently detect the platform’s tilt signals, with the aim of preparing for later mutual comparison and verification to ensure the accuracy and reliability of the measurement results.

The experimental setup includes two self-made ITS sensors and two commercial Guralp 3ESPC sensors, which are placed on an isolation platform inside a thermal enclosure. This setup minimizes the impact of temperature fluctuations and external airflow disturbances on the balance beam. The operational procedures involve calibrating and aligning the sensors, followed by controlled vibration tests and data acquisition using a 16-bit DAQ (Data Acquisition) system operating at a sampling rate of 10 kHz. The experimental verification of the ITS is achieved by comparing the three sets of platform tilt signals among the sensors.

### 4.3. ITS Experimental Verification

We employed differential-mode signals from two commercial Guralp sensors to assess the accuracy of ITS measurements. Unlike the ITS placed directly on the optical platform in Figure 6, we placed two commercial inertial sensors together with two ITS devices on an isolated platform, resulting in three independent measurement systems for platform tilt. By simultaneously recording the platform’s tilt signals with these three sets of sensors, we validated the accuracy of the ITS measurements when the results showed consistency.

**Figure 9:**
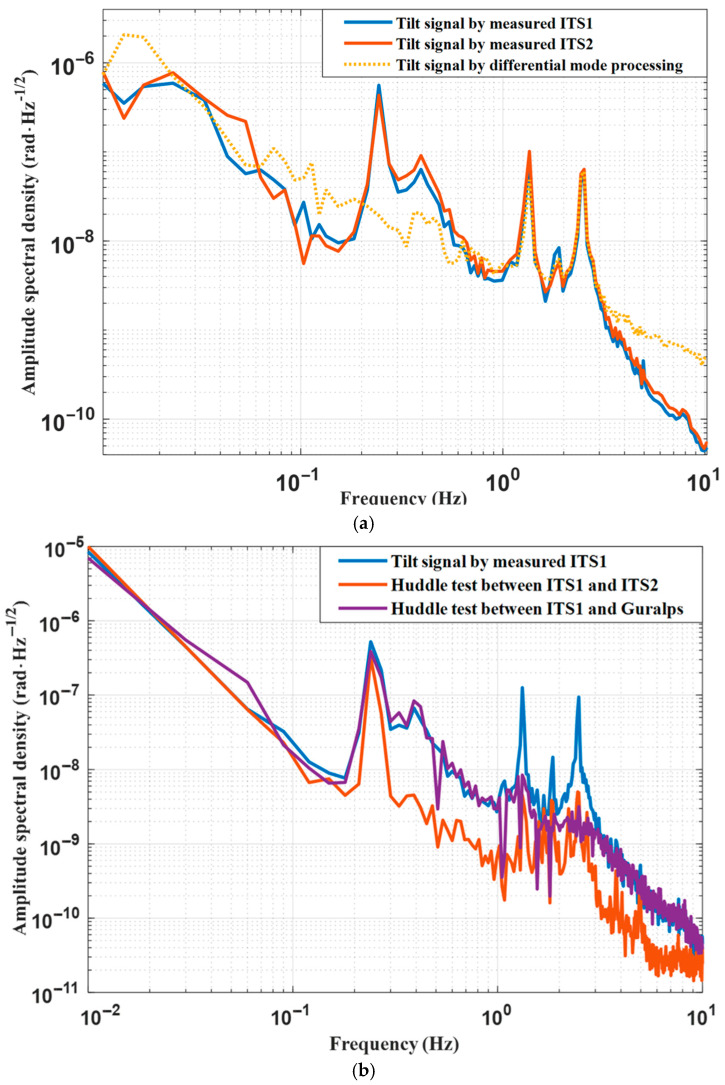

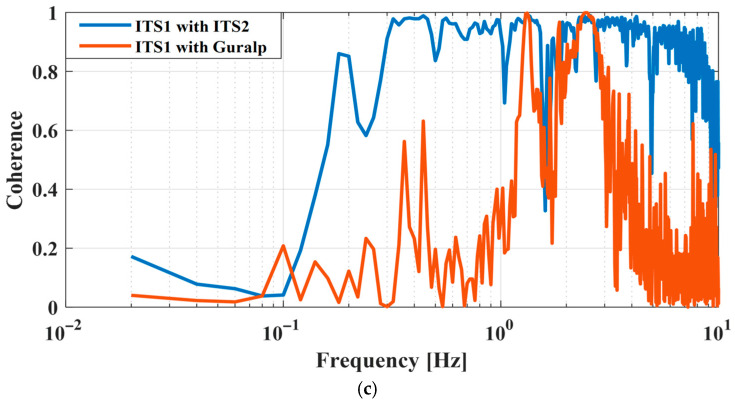
Calibration of tilt signals: (**a**) ASD of the three tilt signals on the platform; (**b**) Huddle test; (**c**) coherence of the ITS1 with ITS2 and Guralp.

Figure 9 illustrates the noise floor comparison, clearly demonstrating the superior sensitivity of the ITS over the commercial sensors. Figure 9a shows the frequency spectra of these three sets of platform tilt signals. The identical frequency spectra observed across all three datasets indicate that the two self-developed ITS equipment measure platform tilt signals with the same fidelity as the commercial sensor within the 0.5–3 Hz range. This similarity in spectral response underscores the accuracy of the self-developed ITS equipment in measuring platform tilt. Nevertheless, at frequencies above 3 Hz, the Guralp differential-mode signals are contaminated by ground noise, rendering it infeasible to measure lower tilt signals. Below 0.5 Hz, the ITS is affected by its resonant frequency of 0.25 Hz, leading to an enhanced sensitivity and discrepancies when compared to platform tilt signals captured by the Guralp differential-mode model. To substantiate the precision of ITS measurements within this frequency range, we analyzed the outcomes from the two ITS devices that showed matching amplitude–frequency curves over the full spectrum of frequencies. This evidence suggests that both ITS sets respond identically when excited by the platform signals, demonstrating excellent correlation at frequencies above 0.2 Hz.

The huddle test involves subtracting non-coherent signals from two coherent signals; this is conducted under the assumption that these signal represent the sensor’s noise floor [28,31]. As Figure 9b illustrates, above 0.25 Hz, the ground signals from both ITS sets are an order of magnitude below the tilt measurement signals, indicating strong coherence. Combined with the analysis of the ITS noise budget in Figure 6, this further illustrates that the noise floor of the ITS is better than that of the differential-mode method when using comparable equipment. Figure 9c shows the correlation of the ITS1 signals with ITS2 signals and Guralp differential-mode signals. It can be seen that above 0.2 Hz, the two self-made ITS devices have good correlation, indicating that they can measure more accurate tilt signals at the same time and have good interchangeability. Below 0.2 Hz, the correlation decreases rapidly, which may result from differences in thermal noise. The correlation between the ITS1 signal and the Guralp differential-mode signal is relatively weak, primarily due to the significant difference in their respective signal noise floors.

The data acquisition system used in this study is the SCALEX10 LabBox-DS6221, which is a high-resolution system capable of sampling at 10 kHz, allowing for the accurate measurement of tilt signals in the desired frequency range.

## 5. Conclusions

This paper introduces and evaluates two methodologies for measuring platform tilt signals: the differential-mode method and the balanced beam method using the interferometric tilt sensor. Both approaches combine theoretical analysis with practical implementation, with a special focus on the ITS developed through the balanced beam method. The ITS’s mechanical structure and interferometric readout system achieve a resolution of 1 nrad$/\sqrt{\mathrm{Hz}}$ at 0.1 Hz and 0.1 nrad$/\sqrt{\mathrm{Hz}}$ at 1 Hz, demonstrating its capability for high-precision tilt detection. Noise assessments showed that thermal-mechanical noise dominates below 0.25 Hz, while ground vibrations significantly impact the measurements from 0.25 to 10 Hz.

The experimental verification phase included setting up both the ITS and commercial sensors on a passive isolation platform for comparison, confirming that the ITS matches the accuracy of commercial sensors within the 0.5 to 3 Hz frequency range. However, the performance of the ITS is challenged at higher frequencies by ground noise and at lower frequencies by resonant effects. Through huddle tests and correlation analysis, we validated the ITS’s minimal self-generated noise contributions, ensuring reliable tilt measurements. Despite the high resolution achieved by the ITS, there are limitations such as sensitivity to low-frequency seismic vibrations and thermal noise. Future work should focus on addressing key challenges such as improving precision, reducing noise, enabling multi-directional measurements, mitigating low-frequency resonant effects, and extending the effective measurement range to higher frequencies. These advancements will refine the ITS’s performance and broaden its applications in gravitational wave detection and vibration isolation technologies.

## Data Availability

Data are contained within the article.

## Abbreviations

The following abbreviations are used in this manuscript:

ITS	Interferometric Tilt Sensor
QRS	Quartz Rotation Sensor
BRS	Balanced Rotational Sensor
LIGO	Laser Interferometer Gravitational-Wave Observatory
aLIGO	Advanced LIGO
ADC	Analog-to-digital converter
ASD	Amplitude Spectral Density
LVDTs	Linear Variable Differential Transformers
DAQ	Data acquisition
PD	Photodiode
PBS	Polarizing beam splitter
CC1	Corner cubes
